# Pittcon 1992 — Abstracts

**DOI:** 10.1155/S1463924692000257

**Published:** 1992


Journal of Automatic Chemistry, Vol. 14, No. 4 (July–August 1992), pp. 113–135

### Pittcon 1992 – Abstracts

The Pittsburgh Conference was held from 9 to 12 March 1992 in New Orleans, USA. The conference included over 400 presented posters and around 1000 lectures. Edited abstracts which relate to laboratory automation are published here. The authors of the most pertinent papers have also been invited to submit full texts for consideration to the *Journal of Automatic Chemistry*.

### NIST CAALS – MODULARITY AND COMMUNICATIONS FOR ANALYTICAL CHEMISTRY INSTRUMENTATION

*Gary W. Kramer, National Institute of Standards and Technology (NIST), Chemical Science and Technology Laboratory, Chemistry Building 222, M/S A-343, Gaithersburg, MD 20899*

The Consortium on Automated Analytical Laboratory Systems (CAALS) has been formed with interested parties from the private sector and other government agencies at NIST to foster the creation of automated chemical analysis systems that will improve the quality of analytical data, facilitate the entire analytical process, and promote the standardization and transfer of analytical methods, while providing industry with competitive advantages in chemical measurement technology.

As one of its initial objectives, CAALS has undertaken, with guidance from its members and others in the analytical instrumentation community, the task of identifying, defining, and promoting general guidelines and standards in critical areas of sample, data, and control information interchange for analytical instruments. Application of such standardized communications methods, together with a modular instrument concept, can dramatically ease the task and lower the expense of fabricating automated analysis systems. Over the past year, the CAALS Modularity Working Group has made substantial progress toward these goals. A logical model for an automated analytical system has been defined, a data model and modelling technique selected, the definition, requirements, specifications, and core commands for standard laboratory modules delineated, and the requirements for two transport links based on the ISO OSI communications model identified.

### ANALYTICAL DATA INTERCHANGE AND STORAGE STANDARDS (ADISS) – PROJECT STATUS UPDATE

*Rich Lysakowski, Digital Equipment Corporation, Four Results Way, Marlboro, MA 01752-9122*

The ADISS Program (ADISS stands for 'Analytical Data Interchange and Storage Standards') has resulted in an industry-wide strategy, methodology, and set of technologies for standardization of analytical data for all major analytical instrument techniques commonly used today. Great progress has been made over the past year in the widespread adoption of this unified, global framework for standardizing analytical data.

The ADISS technologies include various concepts, data models, an application programming interface, data dictionaries, and standards specifications. They are a set of open system standards for data sharing that are independent of any operating system, application, hardware, or vendor.

Scientists (and other end users) receive direct benefits because ADISS provides consistency for analytical data semantics and interchange mechanisms. They ultimately get data interoperability between applications. Instrument vendors and software developers receive the same direct benefits, plus increased programmer productivity because of software reusability, extensibility, and maintainability. Those benefits come from a standardized approach to data interchange and storage. Consistency in data description and access software will eventually increase code and people sharing across the industry, and help reduce time-to-market and development costs.

The ADISS project has made some significant advances over the past year. It has helped foster the creation of public domain, standard ADISS Analytical Information Models (ADISS AIMs) that are being used in the Analytical Instrument Association's (AIA) Generic Chromatography Data Interchange standard, the American Society for Mass Spectrometry's generic mass spectral data interchange standard, the American Vacuum Society's generic surface science data interchange and storage standard. The ADISS AIMs are being ballotted by the ASTM now and have been submitted to ISO.

A public domain ADISS Toolkit and Application Programming Interface (ADISS API), called the netCDF data interchange system, has been recommended to and chosen by many of the above organizations. netCDF is being used for implementation of the ADISS Data Dictionaries for chromatography, mass spectrometry, and surface science. Various vendors are using netCDF in released commercial products now. Many more vendors are getting ready to adopt it for other analytical techniques, including infra-red, atomic absorption, inductively coupled plasma, nuclear magnetic resonance, and UV-VIS spectroscopy.

Technical details of the ADISS Architecture, the ADISS AIMs, the ADISS/netCDF Toolkit, and ADISS Data Dictionaries were explained. Examples on how the standards are being implemented and used in current commercial and non-commercial applications using the ADISS/netCDF approach were given and the presentation concluded with a discussion of where the ADISS Project is progressing.

### THE AIA STANDARD FOR COMMUNICATION OF CHROMATOGRAPHY DATA AMONG COMPUTER SYSTEMS

*David C. Nelson, AIA Committee on Communications Standards, 855 Hamilton Ave, Palo Alto, CA 94301; Michael Duff, AIA, 225 Reineke Lane, Suite 625, Alexandria, VA, 22314; and Vince Dauciunas, Hewlett Packard Corp., 1501 California Avenue, Palo Alto, CA 94304*

In 1988 the Board of Directors of the Analytical Instruments Association (AIS), a branch of SAMA, the Scientific Manufacturer's Association, formally recognized the need for a standard for communication of analytical data among computer systems. The Association commissioned a study on whether such a standard should be created by a technical group from AIA member companies.

In October of 1989 the AIA Standards Committee was formed. The first standard, for the technique of chromatography, has been completed and distributed to end-users and chromatography equipment vendors.

The standard has been tested by the companies participating on the committee, and by a selection of end-users chosen for their work in chromatography and interest in this standard.

A description of the standard, including five categories of chromatography data, key word definitions, and a data interchange system for formatting, was presented, as well as the compiled results of the end-user review phase of the development process. Plans for the AIA Standard release, support and implementation by analytical instrument companies were presented. Finally, perspectives on the ways in which the Standard might be promulgated as it becomes more widely used were given, as well as speculations on the expansion of the standard to other analytical techniques (IR, GC/MS, etc.).

### AN INTERCHANGE STANDARD FOR MASS SPECTROMETRIC DATA

*David Stranz, Scott Campbell, Fisons Instruments, 809 Sylvan Avenue, Suite 102, Modesto, CA 95350; Ray Christopher, Finnigan MAT, 355 River Oaks Parkway, San Jose, CA 95134; James Watt, P-E Nelson, 10040 Bubb Road, Cupertino, CA 95014; and Donald Zakett, Dow Chemical Company, Analytical Sciences Laboratory, Building 1897, Midland, MI 48657*

A proposal for a specification permitting computer-based interchange of mass spectrometric data among instruments of differing manufacture has been developed. Members of the mass spectrometer manufacturer and user community have cooperated in this effort over the past several years.

This interchange proposal is based on the Analytical Instrument Association's Draft Generic Chromatography Data Standard, with extensions to support mass spectrometric data, and the public domain netCDF (network Common Data Format) mechanism for platform-independent data access, developed by the Unidata Program Center.

The mass spectrometry information model describes single- or multiple-scan, selected ion detection, and spectral library data. Most common spectrometer inlets, ionization modes, detection methods, and sampling parameters are included for both low and high resolution instruments. Description of more complex mass spectrometric experiments, particularly FTMS and multidimensional MS, will be incorporated into a future revision of the specification.

The information model provides for sample and sample preparation data, description of the instrument, experimental methods, raw data, processed results, and calibration information. The current proposal treats the first five of these in some level of detail, but defers the processed results and calibration information categories. The overall goal is a complete description of mass spectrometric analyses, sufficient for Good Automated Laboratory Practices and ISO-9000 compliance.

### THE LABORATORY AUTOMATION STANDARDS FOUNDATION: A NEW SERVICE ORGANIZATION FOR LABORATORY STANDARDS

*Joe Liscouski, Laboratory Automation Standards Foundation, Groton, MA 01450*

The use of computers has been a factor in laboratory automation for over two decades. In today's world it would be difficult to consider laboratory automation without computing systems, robotics, networks, and information management systems.

While the individual pieces of a computer-assisted laboratory operation exist, they have yet to function as an integrated system on a routine basis. The situation is more like a puzzle whose pieces do not match and require skilled individuals to make each piece work as part of the system that work is expensive, is often custom, and has to be repeated for each laboratory situation.

A critical part of the answer to 'making the pieces fit' lies in the development of standards for data interchange between systems. Computing networks have been in use for over 15 years, yet it is still difficult to get an instrument data systems and information management systems to work together. Software packages use different data storage formats, which inhibit your ability to move and work with data. Solving those problems today requires the development of unique translators which must be supported and tested; again it is an expensive process.

Once those pieces have been made to fit, one must keep gaps from forming. The technology that computing, instruments, and robotics is built upon is changing, and that change can be difficult to manage. As new products replace those in use, systems that were once working can falter and require modifications and new development work to successfully incorporate the new components.

The solution to these problems is the development and support of standards tailored for laboratory work. That works needs be done by an organization that is able to function freely, not encumbered by proprietary interest, in direct support of end-users, and in partnership with vendors.

The LASF intend to work co-operatively with other standards groups, consortia, end uers, and other organization. Its value to the scientific community comes from the following activities: *standards development & compliance testing services; training courses; reference services; and technology evaluation for laboratory systems.*

---

### ASTM E49.52 COMMITTEE ON COMPUTERIZATION OF ANALYTICAL SCIENCES DATA

*Rich Lysakowski, Digital Equipment Corporation, Four Results Way, Marlboro, MA 01752-9122*

Standards for computerization of scientific data are becoming a major issue for the most large scientific organizations today. Approximately two years ago, three new ASTM Committees were formed to address standards in this area. These committees have gone beyond the hurdles of the organizing process, created scopes and objectives for themselves. They have heard many proposals and each have documents going through the balloting process now.

The three committees are: ASTM E49.50 – Physical & Chemical Property Reference Data; ASTM E49.51 – Chemical Structure Information; and ASTM E49.52 – Computerization of Analytical Sciences Data.

The American Society of Testing Materials (ASTM) E49.52 Committee on Computerization of Analytical Sciences Data is making progress defining standards for computerized analytical data, including chromatography, mass spectrometry, IR, UV-VIS, NMR, AA, ICP, X-ray, thermal analysis, surface analysis data. Abstract data modelling techniques are being used to develop generic analytical information models that will push the lifetime of these standards beyond any particular implementation or format. The ASTM E49.52 Committee is specifically addressing standards for data dictionaries, analytical information models, data access interfaces, data types, data syntax, and data compression. The scope of each of the efforts and details of current activites were explained.

---

### NIST CAALS: STRATEGIES FOR GRAFTING MODULARITY ONTO EXISTING INSTRUMENTS

*M. L. Salit, National Institute of Standards and Technology, Chemical Science and Technology Laboratory, Chemistry B-222, Gaithersburg, MD 20899*

The first demonstration of the CAALS Modularity Project will be in the area of inorganic trace element analysis. The demonstration system will be composed of an automated microwave dissolution system developed at NIST, a sample transfer/chemical moderation system using flow injection concepts and hardware, and a commercially available ICP-OES instrument as the analysis module.

The creation of a CAALS compliant module from the dissolution system is straightforward, as complete design and control latitude is available to the system integrator, who is also the module designer. Creation of a CAALS module from an existing noncompliant commercial instrument carries a different set of problems – a set of problems which will arise for system integrators and manufacturers alike through the next generations of instruments.

These problems arise from the inflexibility of operating remotely an instrument designed to operate interactively. Issues as simple as handshaking (a sample is ready for analysis, the instrument is ready for a sample), new event and status reporting abilities, access to hidden internal data structures, fault tolerance/detection/recovery 'robot friendliness', and support of the CAALS data communications standards will all require addressing.

The discussion focused on the strategies for handling these issues and others which arise in the creation of a CAALS compliant module from the commercial subsystems being used in the demonstration project. General solutions for the use of existing systems within CAALS were discussed.

---

### DETERMINATION OF TRACE METALS IN SOLID SAMPLES VIA ON-LINE DIGESTION FLAME ATOMIC ABSORPTION SPECTROMETRY

*Thomas J. Gluodenis, Jr and Julian F. Tyson, Chemistry Department, University of Massachusetts, Amherst, MA 01003*

A double-flow injection manifold incorporating a resistively heated oven has been developed for the on-line digestion of solid samples followed by direct introduction to the spectrometer. The determination of copper in cocoa powder by flame atomic absorption spectrometry (FAAS) was selected as a model analytical system. The cocoa powder was slurried in 10% nitric acid, injected into the manifold, merged with a stream of concentrated acid and digested under stopped-flow conditions. Gas-liquid separation was effected by a two-stage depressurization system. Copper was determined by flow injection flame atomic absorption spectrometry against acid matched standards. A value of $44 \pm 19$ mg kg$^{-1}$ was obtained. The cocoa powder was also digested using an open-vessel hotplate method and a closed-vessel microwave digestion method. The results were $52 \pm 2$ mg kg$^{-1}$ and $50 \pm 2$ mg kg$^{-1}$, respectively. The relatively large uncertainty in the result for the on-line method was due to the higher dilution in this system producing a concentration in the digest nearer the detection limit of the flame procedure. Subsequent evaluation of the digestion manifold was based on the determination of iron which was found to be present at concentration of 200 ± 10 mg $kg^{-1}$ in the cocoa powder. The proposed procedure increases sample throughput while minimizing sample contamination and decreasing sample and reagent consumption.

---

### DETERMINATION OF ARSENIC IN A NICKEL BASED ALLOY BY FLOW INJECTION HYDRIDE GENERATION ATOMIC ABSORPTION SPECTROMETRY

*C. P. Hanna and J. F. Tyson, Department of Chemistry, University of Massachusetts, Amherst, MA 01003 and S. McIntosh, Perkin-Elmer Corporation, 761 Main Ave., Norwalk, CT 06859*

For the trace determination of elements that form volatile hydrides, hydride generation atomic absorption spectrometry (HGAAS) is a very appealing means of analysis. A problem frequently encountered in HGAAS is the often severe interference effects caused by the presence of transition metals in the sample. Investigators have tried to alleviate these interference effects by complexing or removing the transition metals in the sample matrix, or by optimizing the concentrations of acid an borohydride used in the reduction process. The tolerance for interfering transition metals in HGAAS is greatly improved when it is performed in the flow injection mode. Since this first observation with the discovery of flow injection HGAAS in 1982, investigators have utilized flow injection as a direct means of alleviating these transition metal interferences. However, certain applications have necessitated the use of on-line matrix removal when transition metal concentrations were too high.

In the work presented, the alloy sample is digested with 4% nitric acid/48% hydrofluoric acid in a microwave oven, leaving the arsenic in the sample in the pentavalent state. The enhanced detection limit of the manifold for pentavalent arsenic (0·5 ppb), due to miniaturization of the gas-liquid separator, allows for dilution of the interfering nickel to a tolerable concentration, while leaving the arsenic at a measurable concentration. The manifold has good precision (RSD = 3·5% for 10 ng/ml As(V), $N = 6$), a fully linear calibration over an order of magnitude, and quantitative analytical results for a standard nickel based alloy material (BCS-CRM #346 nickel based alloy As content: Certified = 50·2 ± 3·2 µg/g, Found = 49·6 ± 3·3 µg/g). Manifold optimization strategies for both pentavalent arsenic sensitivity and interference tolerance for nickel were presented.

---

### CLOSED LOOP RE-CIRCULATING REACTORS FOR FLOW INJECTION ATOMIC SPECTROMETRY

*Julian Tyson, E. Grzeszczyk and E. Debrah, Department of Chemistry, University of Massachusetts, Amherst, MA 01003*

Flow-injection (FI) procedures have a number of attractive features, including the possibilities of automation and the provision of a contamination-free sample handling environment. FI manifolds for a variety of pretreatments have been devised but have the limitations that (*a*) solids cannot be handled readily and (*b*) reaction time-scales must be compatible with the normal residence time. To overcome these limitations, closed-loop recirculation reactors and simple valve interfaces have been devised. Calcium has been determined in charcoal by the incorporation of a packed-bed reactor in the loop. The sample was ground (0·5–1·0 mm sieves) and a 40 mg portion placed in a cartridge in the loop. After filling the loop with water, a discrete volume (0·10 ml) of 10% HCl was injected and allowed to circulate for 10 min (0·25 ml total volume). The injection valve then directed a 0·10 ml sample, via a single-line manifold, to the flame atomic absorption spectrometer. Comparison of the results with those obtained by off-line determination of the Ca content showed that 7 ± 1% of the calcium was leached, but as the calcium content is about 4%, the FI procedure has adequate sensitivity, even at the less sensitive Ca line at 239·9 nm. For the determination of Cu in silver, it is necessary to remove the bulk of the silver as unstable silver acetylide is formed in the instrument spray chamber. A loop manifold, containing two injection valves, was used to remove the silver by on-line precipitation as silver chloride. A 1·5 ml sample was injected into a circulating stream (4·0 ml) of 0·5 M HCl. The precipitate was collected on a filter column consisting of nylon fibres packed in a 50 × 3 mm glass column. A 2·5 ml sub-sample was then injected, via the other valve, into a single line manifold for transport to the spectrometer. A recovery of 97 ± 1% was obtained for 10 ppm Cu in 50 g $l^{-1}$ silver. The precipitate was removed by flushing the loop with ammonia solution (1 + 1).

---

### IMPROVING DETECTION LIMITS BY TWO ORDERS OF MAGNITUDE BY COUPLING FLOW INJECTION TECHNIQUES WITH ELECTROTHERMAL ATOMIZATION

*Gerhard Schlemmer, Werner Schrader, Ian Shuttler and Michaela Feuerstein, Bodenseewerk Perkin-Elmer GmbH, PO Box 101164, D-7770 Uberlingen, Germany*

AAS with electrothermal atomization offers absolute detection limits in the lower pg range for most elements. Relative detection limits depend upon, among other factors, the matrix concentration in the samples. Assuming a sample volume of 20 µl, average relative detection limits of around 0·3 µg/l may be obtained for the environmentally and medically most relevant elements. While this is typically more than sufficient to reach the required limits of determination, for example for drinking-water and waste-waters, it is, in many cases, not sufficient to measure the levels of these elements in lakes and oceans. Basically, the same is true for the detection limits obtained with conventional hydride generation techniques although the relative detection limits for elements such as As, Se, Sb, Bi are typically a factor of 5–10 fold superior to those obtained in the graphite furnace.

The flow-injection technique is a very powerful way to automate preconcentration and matrix separation techniques as well as hydride generation. Coupling this sample introduction technique to an electrothermal atomizer opens the way to achieve a new range of detection limits in AAS in an automated on-line system. For most of the measurements, a Perkin-Elmer 4100 ZL AA spectrometer was used in combination with a FIAS 200 system. The FIAS system was equipped with either a column with C18 functional groups bonded to silica for solid phase extraction of metal – DDTC complexes or were set up for hydride generation. The AS-70 graphite furnace autosampler was coupled directly to the flow injection system for the injection of alcohol-eluted metals from the column or to distribute the generated hydrides onto the preheated graphite surface. Pyrolysis and atomization were performed in the conventional way.

Recoveries of close to 100% for both techniques were found for the elements under investigation. Depending on the sample volume preconcentrated on the column or used for hydride generation, the relative detection limit could be lowered by one or two orders of magnitude and could be shifted into the lower ng/l range. Several elements, including Pb, Cd and Cr, were determined in open ocean sea-waters using the solid phase extraction technique and As, Sb, Se and Bi were determined in drinking-waters collected from a large water supply after collection of the decomposed hydrides on the graphite tube. The results were in good agreement with certificate values.

### A MULTIFACET ATOMIC FLUORESCENCE DETECTOR SYSTEM PRIMARILY FOR THE DETECTION OF HYDRIDE FORMING ELEMENTS IN THE SUB PPB LEVELS

*P. B. Stockwell, PS Analytical Ltd, Arthur House, B4 Chaucer Business Park, Watery Lane, Kemsing, Sevenoaks, Kent TN15 5QY, UK; L. Ebdon, W. T. Corns and S. Hill, Polytechnic South West, Drake Circus, Plymouth, Devon PL4 8AA, UK*

The use of vapour generation using continuous flow techniques has become accepted as the only solution where legislative and regulatory pressure has reduced the limits of detection. Professor Stockwell has already shown that detection limits can be improved by a factor of 250–650 using well-defined automated systems.

The coupling of vapour generation techniques with atomic fluorescence to determine mercury has been extremely successful and these devices have become the industry standard in the UK water industry. Extension of the measurement technique into other industries has also become viable, especially for the measurement of low levels of mercury in natural gas and petroleum. The significance in this application being economic and safety, as well as the commonly held environmental and regulatory interest.

Extension of the above techniques for the hydride forming elements has been investigated. Thompson first described a dispersive system for the measurement of arsenic, selenium, antimony and tellurium.

This paper focused on the design criteria for an analytical system to determine the hydride forming elements specifically by AFS. The research work was centred on four areas (*a*) the light source, (*b*) the atom cell, (*c*) the optical configuration of the spectrometer and (*d*) the data collection and processing aspect. Fundamental studies in these areas have produced a simple system designed to provide a flexible and transferable single elemental analysis system.

Detection levels obtained using these developments were discussed and are set out below.

| Analyte | Chemical conditions $NaBH_4$ (% m/V) | HCl (mol $l^{-1}$) | Filter centre wavelngth (nm) | Detection limit (3σ) μg $l^{-1}$ |
|---|---|---|---|---|
| As | 1·5 | 3 | 193·7 | 0·05 |
| Se | 1·5 | 4 | 200·0 | 0·05 |
| Sb | 1·5 | 3 | 215·0 | 0·07 |
| Te | 1·5 | 3 | 215·0 | 0·11 |

The instrument has been used for a wide range of applications and results for certified reference materials correlate well with accepted results.

### SOME CONSIDERATIONS ON THE USE OF NEAR INFRA-RED SPECTROSCOPY AND MULTIVARIATE CALIBRATION IN THE ON-LINE MEASUREMENT OF FUEL QUALITY PARAMETERS

*A. Parisi, J. Medina, L. Nogueiras, L. Carbognani and L. De Lima, Departamento de Análisis y Evaluación, INTEVEP S.A., Apartado Postal 76343, Caracas 1070A, Venezuela*

The use of near infra-red spectroscopy and multivariate calibration is rapidly becoming a standard in the on-line measurement of fuel quality parameters. Several papers published in the literature, simultaneously with a rapid growth in the availability of commercial instruments to perform this task, prove this reality. Yet, a great deal of laboratory research needs to be performed in order to assure the reliability of this analytical technique in a process environment. This paper is aimed to discuss different spectroscopic data treatment schemes and their influence on the robustness of the calibration models as well as the exploration of new applications of near infra-red in the refining industry.

Due to its speed of analysis and low cost compared to traditional methods, the most appealing application of near infra-red spectroscopy is the on-line determination of octane number in gasolines. In spite of the success obtained in measuring the above fuel quality parameter in unleaded fuels, its application to the analysis of leaded ones has been unsuccessful due to the lack of sensitivity that near infra-red has toward the presence of alkyl lead compounds. To overcome this problem the lead content was fed to the calibration model as an extra variable. The obtained results showed the validity of such approach in the determination of octane number in leaded gasolines.

Another fuel quality parameter directly related to the performance of automotive engines is the cetane number. The behaviour of this parameter with respect to the chemical composition of a gasoil or diesel can be easily determined through the near infra-red spectrum. We have proven that the cetane number in a wide variety of this family of fuels can be predicted through the use of multivariate statistics.

Due to a growth of environmental awareness, the measurement of aromatics and olefins content in medium distillates has become of great importance to the refining industry. Yet no reliable, simple, rapid, and inexpensive technique is available to measure this family of chemical compounds in the above matrices. To avoid the use of very time consuming analysis we have explored the possibility of originating a training set made of synthetic standards. Such synthetic standards were made by the dilution of well characterized refining products containing a high concentration of aromatic and olefinic species.

Finally, background correction routines such as single and two point subtraction, as well as second derivative, have been implemented in our laboratory. They all showed advantages and drawbacks, however none of them showed dramatic improvement in the robustness of the calibration model.

---

### AUTOMATED ANALYSIS OF VOLATILE ORGANIC COMPOUNDS (VOCs) OUT OF WATER

*J. Schröder and J. Th. Cornet, Chrompack int. B.V., PO Box 8033, 4330 EA Middelburg, The Netherlands*

The growing importance of detecting VOCs in extremely low concentrations (ng/l, µg/l) and the growing amount of water samples to be analysed make it necessary to automate the very powerful Purge and Trap technique.

During the presentation, two systems, both based on the Purge and Trap technique, were described:

(1) An off-line automatic Purge and Trap system: This system allows 30 samples to be handled automatically.

(2) An on-line automatic Purge and Trap system: This is a fully automated monitoring system that takes samples out of a water source (rivers, lakes, sea, water storage tanks, ring circulation systems etc.) and analyses them using the Purge and Trap technique.

---

### CONTINUOUS WATER MONITORING SYSTEM FOR THE ANALYSIS OF ORGANIC POLLUTANTS IN PROCESS COOLING WATER

*Matthew Przybyciel, James Behm, Tom Sampey and Wanda Gillespie, ES Industries, 701 South Route 73, Berlin, NJ 08009-2621*

Both non-contact and contact cooling water are widely used by the chemical manufacturing industry. In addition, other production and testing facilities, such as jet engine testing, utilize large amounts of cooling water. In a number of instances this cooling water is discharged directly into public waterways where EPA guidelines for levels of organic pollutants prevail. The acceptable levels for many of these pollutants are in the low ppb range and composite samplers provide little more than historical information. It is highly desirable to obtain real time measurements of organic pollutants contained in water discharges.

In this presentation, the development and application of a continuous water monitoring system for the analysis of process cooling water was discussed. The monitoring system is capable of analysing individual chemicals such as trichlorofluoromethane, benzene, trichloroethylene, and methylene chloride at ppb levels using a continuous gas sparging technique and gas chromatography (GC). In addition, chemicals such as ethylene glycol and hydraulic fluids are analysed at ppm levels using GC with direct liquid injection.

Data showing the limits of detection, linearity, precision, and stability was shown. In addition, maintenance and sample filtration considerations were discussed.

---

### THE DEVELOPMENT OF A CONTINUOUS MULTIPLE POINT AIR MONITORING SYSTEM FOR USE IN CHEMICAL PRODUCTION FACILITIES

*Matthew Przybyciel and James Behm, ES Industries, 701 South Route 73, Berlin, NJ 08009-2621*

Both industrial hygiene and environmental requirements have facilitated the need for continuous air monitoring of organic contaminants. These requirements are particularly acute in chemical production facilities where many of the chemicals used and produced are regulated by OSHA. In this presentation, the development and application of an air monitoring system based upon a process gas chromatograph was discussed. This air monitoring system has been used for the analysis of chemicals such as ethylene oxide, tetramethyl tin, carbon disulfide and hydrogen sulfide. Data showing the limits of detection, precision, linearity and stability for these chemicals was shown. The special design considerations, such as explosion proof detectors, electrical classification requirements, and corrosion protection for chemical production facilities was also discussed. In addition, multiple point sampling system design and control, as well as maintenance requirements, were explained.

---

### THE ANALYSIS OF VOLATILE ORGANIC COMPOUNDS (VOCs) IN AIR BY USING MANUAL AND AUTOMATED THERMAL DESORPTION SYSTEMS

*J. Th. Cornet, Chrompack Int. B.V., P.O. Box 8033, 4330 EA Middelburg, The Netherlands*

The determination of VOCs in alrady low concentrations

(ng/m$^3$, μg/m$^3$) in air is extremely important. The only technique for reaching these trace levels is the Thermal Desorption technique combined with capillary gas chromatography.

In this presentation two systems were described.

(1) A manual thermal desorption system: In this system a tube filled with suitable adsorbents is placed in an oven part. The VOCs present on the adsorbents are thermally desorbed into a cryogenically cooled fused silica cold trap. Injection into the analytical capillary column is done by flash heating of this cold trap.

(2) A fully automatic, on-line thermal desorption system: This is a fully automated monitoring system that takes its own air samples out of the environment and analyses these samples automatically using the above mentioned technique.

---

### CONTINUOUS MONITORING OF AROMATIC HYDROCARBONS IN THE ATMOSPHERE AT PPT LEVELS VIA GC

*J. N. Driscoll, C. Wood and M. Whalen, HNU Systems, Inc., 160 Charlemont St, Newton, MA 02161*

Hydrocarbon contamination in the atmosphere is a serious problem since the potential sources are widespread and many of these compounds are involved with photochemical smog formation. In urban areas several hundred different hydrocarbons have been identified by GC at the low to sub ppb levels. This work described the trace analysis down to ppt levels of benzene, toluene, and xylenes in the atmosphere using a portable GC equipped with a PID. The volatile compounds were collected on a trap and thermally desorbed onto the column for analysis. Detection limits of low ppt were realized via this concentration technique and the linear range extended to ppm levels. The system is completely automated for continuous air monitoring and can be set up in the field to eliminate complex sample collection and transfer. Capillary columns were used to eliminate peak overlaps. Details of this automated system, as well as trends observed for BTX in the morning and evening traffic, were discussed.

---

### DESIGN CONSIDERATIONS FOR AN AUTOMATED ON-LINE AIR SAMPLING SYSTEM

*G. M. Broadway and E. A. Woolfenden, Perkin-Elmer, Maxwell Road, Beaconsfield, Buckinghamshire HP9 1QA, UK*

The measurement of volatile organic compounds (VOCs) in the air, derived from factory and automotive exhaust emissions, presents the analytical chemist with a number of problems. The pollutants, which are present at ppb level, must be collected, concentrated, separated and identified. This paper described the considerations for the design of a fully automated system using the Model ATD 400 thermal desorption system for continuous air monitoring.

The trapping of VOCs, below $C_5$ is normally performed at sub-ambient temperatures. This requires large amounts of liquid cryogens which are expensive and may be difficult to obtain at field sites. By using a Peltier cooled trap filled with carbon based adsorbents it is possible to retain the $C_2$ hydrocarbons at −30 °C thus eliminating the need for liquid cryogens. The volatiles are subsequently released into a gas chromatographic column by heating the trap at a controlled rate of 40 °C/s.

The gas chromatographic separation is also a critical part of the system. A multidimensional capillary system has been developed which employs a methyl silicone capillary column and an $Al_2O_3$ PLOT column in series. This approach enables the 60 components listed in the USEPA Atlanta field studies to be separated in a single run, again without requiring sub-ambient cooling of the GC column.

The multidimensional system enables the separation to take place simultaneously on two columns, thus reducing the analysis time, with a subsequent improvement on the sampling frequency.

---

### A NEW QUANTITATIVE INJECTION SYSTEM FOR LABORATORY AND ON-LINE CAPILLARY GAS CHROMATOGRAPHS

*P. Roussel and M. Beche, Rhône-Poulenc Industrialisation, Centre d'Industrialisation de Décines, 69151 Décines-Charpieu, France*

The problem which still limits the development of capillary chromatography in on-line process control is the sample introduction.

The stream-splitting system which is the sole system used today in on-line process capillary gas chromatography (PCGC), does not guarantee the linearity of the splitting ratio and qualitative and quantitative segregation may occur, depending on the nature of the mixture to be analysed and the conditions of the operation. In addition, the loss of apparent sensitivity due to this system is detrimental for trace analysis.

A new injection system, fully automated, has been especially devised for the use in on-line PCGC, but it can also be set up on any laboratory chromatograph. Called 'BIP System' (BECHE Injection Process), it fulfils all the requirements of capillary chromatography. The BIP System allows injections at concentrations of any order of magnitude compatible with the narrowest capillary columns. The system performs fast and narrow plug injections which keep the full column efficiency and better apparent sensitivity. It suppresses qualitative and quantitative segregation. Finally, the repeatability of the actual BIP System is satisfactory, better than 2%.

---

### COMMUNICATION TECHNIQUES FOR PROCESS GAS CHROMATOGRAPH NETWORKS

*Robert A. Williams, The Foxboro Co., East Bridgewater, MA 02333*

The modern Process Gas Chromatograph is a sophisticated, microprocessor-based instrument, capable of performing composition analysis of complex process streams and continuously reporting the analyser results and status to a local or remote station. The need for extensive quantities of validated data from multiple analysers for direct process control, Statistical Process Control (SPC), Statistical Quality Control (SQC), material balance, and data reporting in a Distributed Control System (DCS) is well known. In addition to process control and data reporting some analyser configuation functions may be required from the DCS or maintenance station to insure maximum analyser performance. Monitoring of analyser performance characteristics requires even more extensive communication capabilities. An Analyser Management Station (AMS), utilizing a personal computer, can be used to monitor, control and configure these analysers from a remote location independent from the DCS or analyser shelter.

A network configuration capable of communicating with a large number of GCs to a Network Interface Unit (NIU) was described. The NIU in turn provides a conduit for data direct to the DCS for process control and at the same time allows passthrough of individual analyser messages between the AMS and any analyser on the network. The AMS provides complete analyser maintenance monitoring and configuration from a remote location independent from the DCS. In addition, the AMS computer will display and store chromatograms, gather history data for trend graphs, generate a variety of printer reports, edit analyser control tables and report analyser status and current data.

---

### DEVELOPMENT OF A SIMPLE, LOW-COST SAMPLE ACCESS FOR ROBOTIC APPLICATIONS

*Gerald L. Hoffman and Juliana W. Fahy, US Geological Survey, National Water Quality Laboratory, 5293 Ward Road, Arvada, CO 80002*

A general-purpose moving shelf system was developed to facilitate the movement of sample containers in and out of the access area of a robotic arm and gripper. By moving shelves in and out of the access area, a larger number of sample containers can be processed with a robotic system. The moving shelf system was constructed by modifying a commercial cabinet drawer module. This module was simple to construct, and the cost was less than $200. Movement of the shelf is controlled by the robotic arm. To access the sample containers, the robot arm pulls the shelf out. To clear the area or access a second shelf, the robot arm simply pushes the shelf back in. A springloaded grip handle is located on each shelf to facilitate positioning in either the in or out position. The commercial drawer system used was selected, because it allows for the changing of vertical drawer spacing with a minimum of effort. It is possible to change the vertical position of a given moving shelf in about 1 minute. This allows for flexibility in the size of containers which can be used and minimum down time when trying new configurations. The most significant advantage of this system is its inherent simplicity and reliability. The details of how this shelf system was interfaced with the robotics system were presented, and a working model was demonstrated.

---

### AUTOMATED ENVIRONMENTAL ION TESTING USING NEW OPTIMIZED FLOW ANALYSIS TECHNIQUES

*Shirley, F., Arment and Richard J. Berman, Alpkem Corporation, 9445 S.W. Ridder Road, Suite 310, Wilsonville, OR 97070*

Flow-analysis techniques have remained relatively unchanged in the past 30 years. This work described the incorporation of technological advances into a flow analyser for routine ion testing.

Advances in polymer technology have resulted in such materials as PEEK and Ethyl Vinyl Acetate. These are used for unbreakable and chemically non-reactive heating and mixing coils and connectors.

Other enhancements include the use of improved membrane technology, reliable tubing connectors, and automatic on-line sample dilution for off-scale samples.

Additionally, the system uses a monochromator-based detector to easily decrease the limits of detection without alteration of the sample to reagent molar ratios.

This work also explored some of the advantages of a single instrument using both flow injection and segmented flow analysis techniques. These include dynamic dilution, low dispersion FIA and sample pretreatment techniques.

---

### IMPROVING LABORATORY EFFICIENCY AND QUALITY BY AUTOMATING QUALITY CONTROL AND QUALITY ASSURANCE PROCEDURES FOR ICP-MS ANALYSES

*David W. Boomer and Pamela A. Moss, Ontario Ministry of the Environment, 125 Resources Road, Rexdale, Ontario, Canada M9W 5LA; and Cindy Anderau, The Perkin-Elmer Corporation, 761 Main Avenue, Norwalk, CT 06859-0219*

Laboratory Quality can be improved by implementing a Total Quality Management program. A Total Quality Management program requires that two important principles become part of the daily operation of the laboratory: quality control and quality assurance. Improvements in laboratory efficiency can be attained by automating these procedures as much as possible.

Quality control is employed at the laboratory bench level. For example, an environmental lab would follow QC procedures while operating instruments to analyse a wide variety of sample matrices including waters, sediments and vegetation. A powerful software program, called QC Expert, has been designed for an ICP-MS instrument. It applies the QC protocol defined by the user. This can involve analysing QC samples at a specified frequency, taking user defined actions when entered limits are exceeded, monitoring sample data to determine if results are unacceptable or even within the instrument's detection capabilities.

Quality assurance procedures are used to confirm that the QC procedures are, in fact, controlling the quality of the process. An example of a software program that will automate these QA procedures is called LQAS (Laboratory Quality Assurance System). LQAS performs its tasks by customizing and integrating various components of a quality assurance system. Methods, Staff C.V.s, Standard Operating Procedures, QC Data Summaries, Assurance Charts, Result Approvals, Archival/Retrieval Functions and Standards Databases are example components of a QA system The LQAS system is linked to the QC Expert system via the QC Data Summary function which is common to both systems.

Details of these software programs, currently being utilized in an Environmental laboratory, were described.

---

### TESTING ANALYTICAL INSTRUMENT SOFTWARE: A CHEMIST'S VIEWPOINT

*Marc A. Tischler and Steve Madden, Hewlett-Packard, Scientific Instruments Division, 1601 California Avenue, Palo Alto, CA 94304*

Chemists rely on computers to executve complex computational tasks and extend their abilities. However, this dependency also demands confidence in the process used to develop the instrument vendor's software. Specifically, the testing phase of the software lifecycle is critical in delivering a reliable and accurate product to the chemist.

The presentation communicated, in chemist's vocabulary, the software testing approaches used. To convey these ideas, an analogy was drawn between software development techniques and standard methods of known chemical components.

For instance, developing a software testplan in R&D parallels designing quantitative chemical analyses that assure the validity of a lab procedure. Also, collecting performance and reliability software metrics corresponds to monitoring the suitability of an analytical instrument by periodically checking against known calibration samples. In software as well as in the lab, meeting the criteria means synthesizing the control information into an optimized procedure which finally becomes stabilized as a software release or standard method.

---

### A STATISTICAL APPROACH FOR THE EVALUATION AND REPORTING OF LIMS DATA

*M. A. Epton and G. F. Gostecnik, Analytical Automation Specialists, Inc., 11723 Sunbelt Court, Baton Rouge, LA 70809*

Often the collection of analytical data is recognized as a costly but necessary function in a chemical process or research environment. The perceived need to gather this valuable data into an easily accessible central repository and maintain it over long periods of time has given rise to the computerized Laboratory Information Management System (LIMS). However, the simple reporting and storage of this data may not be sufficient to meet the needs of those paying for its collection. This paper reported the development of ancillary LIMS software, LABWORKS-SQC, which, through the use of statistical techniques, provides improved quality of measurement along with documentable analytical precision and potentially large time savings in product analysis.

Statistical Quality Control (SQC) is not just a means of improving product quality, but may also be used to improve the quality of the analyses of those products. Employing LABWORKS-SQC, analytical precision is determined by analysing known standards and process capability by statistical evaluation of this data. Control limits are developed statistically for each class of data.

In routine use, sample requests are automatically logged and instrument parameters downloaded. After analysis, if the data are found to be within the previously determined limits, the results may be immediately transferred electronically to the requestor in the form of control charts, formatted reports or certificates of analysis. Feedback to the analyst helps determine the need to recalibrate, reanalyse, or resample. This critical decision-making area represents one of the largest potentials for time savings in the laboratory. Savings are also realized through timely reporting and the need to review only out of limit data.

---

### ENHANCING THE AUTOMATION OF A QUALITY CONTROL LABORATORY

*D. M. Dion and E. J. Citron, Millipore Corporation, Waters Chromatography Division, 34 Maple Street, Milford, MA 01757*

The ability for laboratories to comply with GLP/GMP regulations while maintaining laboratory automation is a necessity within the chromatography industry today. Quality-control (QC) laboratories that perform high throughput separations require advanced data handling techniques while ensuring system ease-of-use. An innovative software package has been designed to afford QC laboratories the ability to comply with regulatory requirements while providing the laboratory with maximum automation from sample login to data summaries to custom reports. The focus of the software is to provide the user with the means to efficiently handle large amounts of data. The software utilizes capabilities which allow the user to simultaneously collect data in real time while utilizing third party software packages or reprocessing/reviewing previously collected data. A discussion of enhancing productivity with networking was provided.

The focus of the presentation was to demonstrate the ease of operation of this software package and its value to a regulated quality control laboratory. The advanced security abilities within this software allow varying degrees of access to be assigned to users. A separation procedure can be developed based on a method template which includes routine method information. This allows the bench chemist to focus on individual sample information when logging in samples. Results are directly accessible by system suitability software allowing quick system validation while decreasing possible transcription errors. Other features include flagging values that fall out of a user defined designated range, the ability to customize data summaries and a report designer format.

---

### GOOD AUTOMATED LABORATORY PRACTICES IN THE MS LAB

*Norman Low, Neerja Raman, Hewlett-Packard, 1601 California Ave., Palo Alto, CA 94304; and Tim Matthews, Thru-Put Systems, Inc., 450 East South St, Suite 201, Orlando, FL 32801*

In December 1990, the US Environmental Protection Agency issued a draft on Good Automated Laboratory Practices (GALP). This document contains recommendations for ensuring integrity in computerized systems. In general, the principles reflect historical standard laboratory practices. What is new for the laboratory is the implementation of these practices in all aspects of computerized operations. Although some of these guidelines have been applied to laboratory information management systems, they have not been used in instrument controllers.

The basic principles for the GALP guidelines are:

(1) Data: Assure integrity of all entered data.
(2) Formulae: Assure that formulae/algorithms are accurate and appropriate.
(3) Audit: Track data entry/edits to responsible person.
(4) SOPs: Use appropriately documented procedures.
(5) Disaster: Provide alternative plans for failures and unauthorized access.

The mass spectrometry group is one of the most computer-intensive centres in an environmental laboratory. One could argue that typical MS target compound analysis could not be done without the computer. However, most MS data systems have been designed for an open environment – any operator can perform any function. Furthermore, changes made in the processed data are not tracked in the way required by GALP.

The responsibility for GALP compliance rests on the laboratory management. They must ensure that the lab's operations are consistent with the guidelines. Much of the work required consists of non-automated checking at various stages and manual operations. These characteristics lead to inconsistencies. Some features can be incorporated into a data system to make it easier to comply. The MS ChemSystem with Target2 software has implemented such items as security through multi-level passwords which determine functional access, password-controlled keyboard lock, integrated method QA, audit trail, protected results files, and automated data backup. Physical security of the computer and the media is possible since the computer can be locked in a cabinet, hundreds of metres from the instrument location. These and other features assist the lab in meeting the GALP guidelines.

---

### SUPPORTING HETEROGENEOUS NETWORKS IN AN ANALYTICAL LABORATORY ENVIRONMENT

*Mary Barnier, Hewlett-Packard Company, Palo Alto, CA 94304*

With the onslaught of networking demands and the huge variety of networking products in today's market to meet those demands, chemists and laboratory managers are faced with many decisions about systems and communication connections. The recent outpouring of network technology products offers the opportunity to make the act of collecting and analysing data from multiple instruments a simplified, automated process.

Typically, in today's laboratories, networking is a very centralized function and only one type of network prevails in all areas. But the industry anticipates rapid expansion in the future and this will require larger and more diverse networks. With this burgeoning demand for networking expansion, chemists and managers of laboratories are going to need to know more and more about how to support these heterogeneous networks on a day-to-day basis.

Networking standards have recently been established to help laboratories make the transition into networking with minimal support overhead. The Hewlett-Packard (HP) Unified Laboratory concept is one such strategy based on one of the most popular and general purpose network standards available. The Unified Laboratory provides chemists with networking products that are consistent with HP's analytical data system user-friendly interfaces, and functionality that is tailored to fit with analytical applications on various platforms from different vendors.

With each new set of networking technology, another door of opportunity is opened for chemists and laboratory managers to continue improving the throughput of their laboratories. However, as analytical laboratories take advantage of the benefits of today's, and tomorrow's, networking technology, it will become increasingly important for these varied communication products to interact. The Unified Laboratory facilitates the connection and expansion of multisystem, multivendor networks that are inherently easy to support.

This paper discussed ideas and methodologies that will help the chemist or network administrator focus their network needs allowing them to conform to compatible network standards like IEE 802.3 and TCP/IP. It addressed how multiple protocol technologies may coexist in a single environment, such as TCP/IP and IPX/SPX. Prevention techniques that will reduce data and productivity losses in these heterogeneous environments were also explained.

---

### PROCESS MONITORING OF MOISTURE IN A FLUID BED DRYER

*Frank A. Dethomas and Jack Carroll, NIRSystems, Inc., 12101 Tech Road, Silver Spring, MD 20904*

A uniform mixture of excipients and actives in powdered materials is obtained by wet granulation process. After the granulator, it is necessary to reduce the moisture level for final handling. The reduction of the moisture level in the product is most commonly accomplished in a fluid bed dryer.

In the processing of powders to become finished products, one of the biggest difficulties is the accurate measurement of the moisture in the fluid bed dryer. Many techniques are available for the laboratory for moisture measurement but few translate to the process. The fluid bed dry applies both dry gases and heat to reduce the moisture level.

This presentation discussed the technical problems with on-line measurements in the fluid bed dryer environment and development of a NIR based on-line measurement in the fluid bed dryer.

---

### AUTOMATED ANALYSES FOR VARIOUS CYANIDE SPECIES IN WATER USING A SINGLE CONTINUOUS FLOW MANIFOLD

*Richard J. Berman and Shirley F. Arment, Alpkem Corporation, 9445 S.W. Ridder Road, Suite 310, Wilsonville, OR 97070*

The measurement of various species of cyanide in water is important when considering their differences in environmental behaviour and toxicity. While small quantities of simple cyanides are extremely toxic, iron complexes are not nearly as toxic.

Current standard manual methods for cyanide analyses are time-consuming, require special glassware and large sample volumes, and generate a great deal of hazardous waste. Current automated methods are complicated, subject to interferences, and do not easily allow for the measurement of multiple cyanide species on a single flow manifold.

A simple, continuous flow manifold has been developed which is capable of determining free cyanide, weak and dissociable cyanide, total cyanide, cyanides amenable to chlorination, and cyanogen chloride, with minimal changeover required between tests. In order to avoid thiocyanate interference, a Pyrex glass reactor was used to prevent photoconversion of thiocyanate to cyanide. In addition, membranes were investigated for separation of the various species of cyanide. Data will be presented showing the results of this investigation on low detection limits, accuracy, precision, and sample throughput.

---

### A MINIATURE MEMBRANE DISTILLATION TUBE FOR RAPID PARALLEL DISTILLATIONS OF CYANIDE, PHENOLICS, SULFIDE, AND AMMONIA IN WATERS

*Scott Stieg, Lachat Instruments 6645 W. Mill Road, Milwaukee, WI 53218*

Despite the availability of rapid, accurate, and specific flow injection, segmented flow, and convenient batch methods for the determination of volatile analytes such as ammonia ($NH_3$), hydrogen cyanide (HCN), phenol, hydrogen sulfide ($H_2S$), most standard methods for these analytes call for a manual pre-digestion and pre-distillation of the sample to recover complex forms of the analyte and to then remove the analyte from a possibly intefering matrix. Continuous-flow analysers sometimes employ a flowing-stream digestion and distillation device in which the sample to be determined is digested and distilled continuously as a front-end extension of the method. Because of their dependence on tricky and maintenance-intensive miniature versions of conventional nineteenth century glassware, these approaches suffer from long start-up and line-out periods, variable recoveries due to the necessary short residence times, and, because of the large on-line volume, sample carryover for realistic dynamic ranges is severe at throughputs approaching that of the continuous-flow method itself.

Lachat has developed a batch microdistillation system, Micro-Dist, as an alternative which does not use conventional phase separators or condensors. 6 ml samples are placed into plastic tubes. The tubes are sealed at the same time a releasing acid or base is added. The sample is then boiled in the tubes in a block heater. Vapour passes through a hydrophobic membrane, condenses above the membrane, and the condensate is collected over the membrane. A trapping solution can be placed over the membrane for analytes such as ammonia, cyanide, sulfide, or phenolics. Loss of water vapour and analyte out the top of the tube is minimized by a second membrane and passive temperature gradients in the collector section of the tube.

100% recovery in less than 60 minutes for samples spiked with cyanide, complex cyanides, sulfide, phenol, and ammonia has been achieved. Since the samples are distilled in parallel, the distillation sample throughput is not limited to the rinse-out period of a continuous-flow method. In addition, even using batch methods for the determination, the throughput, including sample preparation, is increased by removing the need for time-consuming setup and tear-down of conventional distillation glassware.

---

### INTEGRATION OF AN AUTOMATED GPC SAMPLE CLEANUP SYSTEM INTO A STANDARD LABORATORY MODULE

*D. L. Stalling, K. Kelly, D. Lochhaas, J. Upham, A. Tsang, D. Grant, L. Cernohlavek, ABC Laboratories, Inc., PO Box 1097, Columbia, MO 65205; and B. Wilding, EG&G Idaho, Inc., INEL, PO Box 1625, Idaho Falls, ID 83415-3561*

A Cooperative Research Agreement (CRADA) between ABC Laboratories and the Department of Energy, Idaho

National Engineering Laboratory was formed to integrate a high performance, automated Gel Permeation Chromatography (GPC) sample processing system (AS-2000) as a module in the Department of Energy's Standard Laboratory Module (SLM) for EPA Methods 3550 and 3540. The complete integrated SLM system provides automated extraction, filtration, sample transfer and dilution, separation of co-extractive and biogenic material from contaminants, sample concentration, and aliquoting processed extract. A sample receiving module utilizes ultrasonic sensing in the flow path to ensure accurate sample loading and dual level-sensing to provide for sample dilution. The sample concentration module evaporates solvent and concentrates analytes separated by a high performance Envirosep-ABC GPC column prior to analysis without going to dryness or using a 'keeper'. The evaporation module provides for recovery of spent GPC solvent.

The SLM for these EPA methods is a joint development goal for DOE's Contaminant Analysis Automation Technical Working Group operating as part of the Robotic Technology Development Program 5-year plan. Software design and communication protocols for the exchange of signals and commands between modules composing the SLM and a master control unit are planned to conform to modularity and communications protocols being developed by the National Institute of Standards and Technology's Consortium on Automated Analytical Laboratory Systems. Design and control of the system was discussed in terms of modularity, enhancing automation of sample processing and process control.

---

### ON-LINE COUPLED SFE/HRGC AND MICRO-PREPARATIVE SFE AS A SAMPLE PREPARATION TECHNIQUE

*A. D. Bashall, Fisons Instruments, 32 Commerce Center, Cherry Hill Drive, MA 01923; F. Munari and F. Andreolini, Carlo Erba Instruments, Strada Rivoltana, 20090 Rodano, Milan, Italy*

Supercritical fluid extraction (SFE) has begun to be increasingly popular as an alternative to solvent extraction and/or complicated multiple extraction procedures.

The instrument described (SFE 3000 Analyser) can be operated as an on-line device coupled to a HRGC (or SFC) or as a preparative system employing sample collection. The extraction is carried out in a temperature controlled oven and can either consist of single or multiple samples. The multiple extraction system enables up to six samples to be extracted sequentially. The ability to collect the extract can be important in many cases where non chromatographic analysis is required or where sample clean-up or pre-treatment is required prior to analysis. Careful considerations need to be taken into account when performing on-line SFE, these considerations should address the chromatographic integrity of the sample to ensure no excessive band broadening is taking place. Reconcentration of the sample prior to the chromatography is important and was discussed in detail.

Off-line SFE, enabling a fraction to be collected, must also be considered carefully to ensure the sample is not being altered prior or during the collection process. Different collection mechanisms were discussed: dry, cold trapping, solvent or solid supports.

The SFE technique is also applicable to multi hyphenated techniques such as SFE/GC/MS. Various sample types were discussed relating to a wide range of samples and applications.

---

### AN INTELLIGENT SYSTEM FOR THE AUTOMATION OF USEPA CONTRACT LABORATORY PROGRAM (CLP) REQUIREMENTS FOR ELEMENT DETERMINATIONS IN WASTE WATERS BY ATOMIC ABSORPTION SPECTROMETRY (AAS)

*Michael B. Knowles, Varian Australia Pty Ltd, 679 Springvale Road, Mulgrave 3170, Victoria, Australia; and Fred Delles, Varian OSI, 201 Hansen Court, Suite 108, Wood Dale, IL 60191*

Laboratories participating in the United States Environmental Protection Agency (USEPA) Contract Laboratory Program (CLP) are required to follow stringent quality assurance and control procedures to validate analytical data. The complexity of these procedures requires the analysis of a large number of test solutions, restricts the number of sample determinations and requires close monitoring to ensure compliance.

The aim of this work was to develop a system for the automation of USEPA CLP requirements for the analysis of waste waters by AAS. The software developed allows the operator to specify the frequency of measurement of a QC Standard, the upper and lower limits of the result and the action to take if outside these limits. The most rigorous action is to recalibrate, re-run the QC Standard and if this passes, re-run all samples since the previous correct QC Standard.

Similarly the operator can specify the frequency of measurement of a QC Spike. The software automatically calculates the spike added concentration. Upper and lower recovery limits are specified, together with a minimum limit.

An over-range volume reduction facility automatically reduces the volume injected by the specified factor. The volume is continuously reduced until the solution is in range or a limit of less than 1 μl is reached.

When a spike is performed, the sample and its spike must be run as a consecutive pair. When an over-range sample is encountered the system acts to produce an in-range result. The sample is then spiked and if over-range, the sample volume is reduced and the sample re-run. The new (smaller) sample volume is then spiked and the solution measured, ensuring that the sample and spike are determined as a pair.

Six other tests are also performed on the data and all solution data is time and data stamped.

---

### INTRODUCTION OF A NEWLY DEVELOPED LOW COST AUTOMATED FLAMELESS ATOMIC ABSORPTION SYSTEM FOR MERCURY ANALYSIS

*Peter J. Kmieck, Kappa Laboratories, Inc., 7019 SW 13th Street, Miami, FL 33144; Marlin Bensinger, Chromtec, 7544 Lake Worth Road, Lake Worth, FL 33467; and Herb Kenny, LDC Analytical Inc, 3661 Interstate Park Road N., Riviera Beach, FL 33404*

Mercury, and in particular, organosubstituted mercury moieties have been recognized as a potential cumulative health threat emanating from several sources. The number of samples and replicate runs required makes automation of this analytical method an attractive and economical option available to regulatory laboratories. This traditionally involved dedicating an expensive multipurpose analyser to the mercury analysis.

The new LDC Analytical automated mercury analysis system incorporates an autosampler, programmable bubbler module, new high sensitivity photometric detector, and data acquisition system.

The new system is modular in design, can be operated manually in a research/set-up mode or fully automated as needs vary. Data on CV and MDL were discussed, with comparisons made to existing methodologies. The automated system was compared to the conventional manual process of analysis previously employed. Containment, execution of reaction sequence and data collection were discussed.

---

### A DEDICATED PURGE AND TRAP/GC SYSTEM FOR ENVIRONMENTAL ANALYSIS

*J. W. Washall, T. P. Wampler and W. A. Bowe, CDS Analytical, Inc., 7000 Limestone Road, PO Box 277, Oxford, PA 19363*

The growth in the field of environmental analysis over the past 10 years has placed an emphasis on complete systems designed to perform a number of environmental procedures. Purge and Trap systems interfaced to gas chromatographs can sometimes be rather bulky systems involving lengthy transfer lines which uses up bench space. This paper described a purge and trap system which has a self-contained gas chromatograph for dedicated environmental applications.

The focus of this study was to demonstate linearity, reproducibility and recovery for various environmental analysis procedures. The system is capable of purge and trap analysis of water and soil samples as well as the ability to thermally desorb air monitoring cartridges. Samples are processed in the purge and trap system and then separated with the internal capillary gas chromatograph.

Providing a compact dedicated purge and trap/GC system reduces the amount of bench space needed while providing highly sensitive and accurate analysis. The GC is capable of performing without using the purge and trap system and can be equipped for both split and splitless injection. The chromatographic oven can be programmed up to 400 °C. Samples analysed in this evaluation include water, soil and air monitoring cartridges.

---

### MULTIMETHOD AUTOMATION FOR ENVIRONMENTAL EXTRACT CLEAN-UP

*Mark Cava and John Helfrich, Zymark Corporation, Hopkinton, MA 01748*

Traditional extract clean-up methods require manual dilutions, filtrations, injections, florisil cartrige conditioning, sample loading, and reagent additions. This presentation discussed:

(1) Automated environmental extract clean-up methods.
(2) The workstation's automatic log of sample handling.
(3) The GC/MS results for the analytes of interest.

Three different applications of the BenchMate are examined:

(*a*) PCB clean-up.
(*b*) Traditional GPC clean-up.
(*c*) High performance GPC clean-up.

Environmental extracts were analysed for PCBs in oil, semivolatiles, pesticides, and PAHs. The BenchMate workstation performed weighing, dilution, and florisil cartridge/acid clean-up for PCBs and dilution, filtration, and injection for GPC clean-up.

The recoveries of PCB (note: concentrations were less than or equal to 4 ppm) were 80% or better for duplicates.

For traditional GPC clean-up, soil was spiked with 100 ppb of the semivolatile surrogates and pesticides. The two process evaporations (180 ml of primary soil extract was taken to 1 ml and 150 ml of the subsequent GPC collect fraction was taken to 1 ml) were performed in the TurboVap evaporation workstation. Recoveries of semivolatiles and pesticides were between 51% and 73% without the use of any keepers. Recoveries of selected analytes from straight solvent spikes were greater than 85%. The spread between soil extract recovery values ranged from 4·5% to 9·1% for five semivolatile surrogates.

High performance GPC analysis of PAHs had recoveries of 85% or bettter for concentrations at 1 ppm. The spread between recovery values for two determinations range from 1% to 8% for 6 PAHs. The samples were filtered and injected by the BenchMate onto a high performance GPC column. Fractions were collected in TurboVap tubes (50 ml collect volumes) and concentrated to 0·5 ml in the evaporation workstation.

---

### STATISTICAL TOOLS USED IN IMPROVEMENT OF QUALITY CONTROL LABORATORIES' PERFORMANCES

*Mihai Ciopec, Genesis Polymers, a NOVA affiliate, 2550 Busha Highway, Marysville, MI 48040*

Quality Control Laboratories from Genesis Polymers are designed and equipped to assure a continuous quality of polypropylene products: homopolymer, impact copolymer, and ultra high impact TPO copolymer.

To fit all our customer specifications Genesis run more than 75 mechanical, chemical, and physico-chemical tests. Some of these are very critical especially for automotive industry. For this reason a statistical program to evaluate the performances of gages, measuring devices, instruments, methods, and operators was implemented.

The program 'Gage Repeatability & Reproducibility (R&R pack)' is a software package from PQ System. With this program statistical evaluations of the following are run:

(1) Accuracy/deviation from the target of the measured characteristics.
(2) Accuracy/linearity of the observed values versus the true values.
(3) Capability indices of the gage, device, instrument, and of the measurement process.
(4) X bar and range from the control chart of measurement process.
(5) Variability, in terms of measurement variation compared to the process variation.
(6) Equipment and appraiser variation.
(7) Repeatability and reproducibility.

---

### BARCODED LIMS USER INTERFACE

*Claude Gonzalez, Jean-Pierre Philipot, Analytical and Physical Chemistry Department and Rhone Poulenc Rorer, Centre de Recherches de Vitry-Alfortville, 13 Quai Jules Guesde B.O.14, 94403 Vitry-Sur-Seine, France*

Implementating a LIMS for all the Analytical and Physical Chemistry activities (seven departments, 140 people) involved in drug substance research and development is a challenge. It was nevertheless the main objective since maintaining historical information about projects is the most time-consuming administrative job in a GLP/GMP environment.

In order to achieve this, a flexible database management system was purchased but electronic forms were not customized, mostly because of development cost and time. Instead, an interface based on barcoded reports and procedures for logging studies, samples and tasks, entering results or tracking samples was implemented.

Reports are automatically edited to check correct data entry or label samples. They included the barcoded identification number of the study or sample, allowing quick and safe access to data.

Procedures guide the user into the different forms and include barcoded transcription of commands, function keys or data. Only alphanumerical keys of the keyboard are needed to enter sample names, results or text.

Barcoded procedures are specifically adapted to the day-to-day activities of the various departments and ensure error-free data entry, even by unfamiliar users. Thus, acceptance of the system and impact on quality are immediate, and training costs are lower.

The time needed for one operation is decreased by 40 to 90%: productivity is increased, and use of the computer system is optimized.

---

### SELECTING THE ENVIRONMENTAL LIMS

*Scott A. Johnston, Galson Laboratories, 6601 Kirkville Road, East Syracuse, NY 13057*

With the overwhelming array of Laboratory Information Management systems (LIMS) available, it can be quite a formidable task selecting and implementing the right one for an environmental laboratory. Many LIMS systems on the market are not specifically designed for the unique requirements these laboratories face. This paper will discuss what to look for and what to avoid while selecting a LIMS product for the environmental laboratory.

Flexibility is the most important feature of the environmental LIMS. Ever-changing regulations make an open, modifiable system essential for the laboratory to stay current and provide quality service to its customers. The LIMS must allow real-time data collection from all laboratory instruments, perform necessary post analysis calculations and produce the required report format. For example, our LIMS has been configured to collect data over RS-232 lines from GC, GC/MS, ICP, AA and IC instruments. Calculations and QC checking protocols have been entered to produce reports required by the US EPA Contract Laboratory Program. This level of flexibility has allowed the laboratory to reduce reporting errors from greater than 20% to less than 2%. The report package production time has decreased from about two days to less than 2 hours.

A successful environmental LIMS must also automate the real world business situations that require quick, accurate information retrieval. Sample status tracking by department and *ad hoc* queries are essential tools for ensuring quality customer service. Since LIMS, client services personnel are able to answer most customer calls immediatley by performing a simple on screen query.

---

### LIMS RUNNING ON MACINTOSH MICROCOMPUTERS: A POWERFUL TOOL IN ANALYTICAL RESEARCH LABORATORIES

*P. Cleon, Rhone-Poulenc Industrialisation, 24 Avenue Jean Jaures, 69151 Decines-Charpieu, France*

Most LIMS have been designed for laboratory data management in order to acquire and gather information, ensure storage and integrity of data. However, these valuable tools for laboratory management and quality control are not always adapted for non-routine work such as the use of sophisticated spectroscopy techniques or for research.

The objective was to develop a multiuser database on microcomputers giving access to sample management, storage of experimental conditions, validation, data treatment, passwords for confidential information, etc.

In addition to all these capabilities, this easy-to-use software (Macintosh environment) offers unique possibities of information treatment and results edition: (1) word processing combining chemical structures, graphics and spectra; (2) a table generator for rapid production of complex tables (with chemical structures, scientific calculations or statistics); (3) graphics editor for generating curves from experimental data or the database.

The possibility of combining pictures and texts makes this new data management system particularly useful for molecular spectroscopy laboratories.

The results obtained during a two-year evaluation period show that microcomputers can be very powerful instruments in the field of laboratory data management. Results also demonstrated the possibilities, limitations and advantages of this system which turns out to be a very promising tool for research analytical laboratories.

---

### EXPERIMENTAL DESIGN, STATISTICAL, AND DATA QUALITY CONSIDERATIONS IN THE FIELD EVALUATION OF INNOVATIVE ENVIRONMENTAL MONITORING TECHNOLOGIES

*Charles B. Davies and Vicki A. Ecker, Lockheed Engineering & Sciences Company, 1050 E. Flamingo, Las Vegas, NV 89119; Stanley N. Deming, Department of Chemistry, University of Houston, 4800 Calhoun Road, Houston, TX 77004; and Stephen Billets, US EPA Environmental Monitoring Systems Laboratory, PO Box 93478, Las Vegas, NV 89193*

The US EPA regularly tests new technologies in order to identify promising methods for further development and use in Superfund site characterization and remediation. Technical and conceptual challenges abound in the design of these studies, which typically involve limited resources and emerging technologies.

Data Quality Objectives (DQOs) differ from those applied elsewhere (for example for routine analyses performed in the course of a monitoring or remediation effort), as the objective of a technology evaluation study is to determine the level of data quality attainable with the technology being tested. For such a study it therefore becomes appropriate to emphasize DQOs for the design and implementation of the experiment and to modify traditional quantitative DQOs to reflect the goals of the study.

Establishing and meeting such DQOs requires careful attention to both the conceptual and practical aspects of the experiment, including (1) thoroughly contemplating all expected and potential sources of random and systematic variability in the measurements to be made, performing literature searches and/or pilot studies are required; (2) using sample observations efficiently for multiple purposes, such as incorporating QC samples into other aspects of the study; and (3) using paired comparisons and similar techniques for making samples measured by different instruments as nearly comparable as possible, to help control for unavoidable and/or unanticipated modes of variability.

Such careful planning can aid greatly in making the statistical analysis of the resulting experimental data simple and easy to interpret, and in ensuring the scientific soundness of the conclusions. However, time and resource constraints can create inherent limitations on the strength and generality of conclusions drawn from field studies. These should be anticipated in the planning process, and should not be viewed as weaknesses of the study. To the extent that these limitations prevent the technology evaluation study from giving unambiguous approval (or the contrary) to the technology involved, the field study should be regarded as a screening study, to be followed by further evaluation upon conditional approval of the technology or by further research or development and re-evaluation, as indicated by the study.

These concepts were illustrated in detail using the recent performance evaluation of the $A^{+}$RT Volatile Organic Analysis System, an automated sampling and analysis system designed for the continuous monitoring of VOCs in a ground water remediation effort, which is the subject of another paper to be presented at this Conference.

In addition, suggestions for future research on methodological issues related to the design of such studies were presented.

---

### THE ANALYTICAL CHEMIST'S LOGBOOK: AN ELECTRONIC SOLUTION

*Antoinette M. Burkett and Gary Powers, Hewlett-Packard, Scientific Instruments Division, 1601 California Avenue, Palo Alto, CA 94304*

Currently many chemists go through the daily drudgery of writing entries into a paper logbook recording sample statistics. A typical sample logbook may include such things as date, time, job number, sample identification, bottle number, and operator. Often the information recorded by hand already exists in the data system software. This paper will discuss how the information can be accessed automatically using the HP-UX ChemSystem software. Using these techniques, a report can be generated automatically, or if preferred, read instantly into a third party application. This allows complete electronic automation of the logbook entry process.

Information that can be made available to third party software is not limited to sample logbooks. Following the same procedures, custom report information such as QA Summaries using recovery percentages and relative percentage differences can also be made available to third party applications.

By using UNIX-based analytical ChemSystems the chemist can take advantage of network capabilities which allow the third party application to exist on any of several networked systems. Data from each system can be extracted and recorded in the central spreadsheet database. Techniques by which data can be extracted and transferred from UNIX ChemSystems to third party applications were described using a sample logbook and QA summary report as illustrations.

---

### COMPUTER TECHNIQUES FOR VISUALIZATION OF ANALYTICAL DATA

*Mark Glick, Paul J. Galley and Gary M. Hieftje, Department of Chemistry, Indiana University, Bloomington, IN 47405*

A new collection of computer methods for extracting information from multivariate data has become available to analytical chemists – data visualization techniques. Visualization techniques can transform multidimensional data into graphical representations that can be manipulated by the scientist. The use of sophisticated graphics, object rendering techniques, animated display techniques, colours, and other graphics procedures can greatly enhance the apparent information content. These methods allow scientists to examine multidimensional data in geometrical format (for example as pictures) rather than in numerical format.

Visualization techniques are most useful when an abundance of multivariate data is available from analytical measurements. One example in our laboratory is the study of the inductively coupled plasma for diagnostic purposes. Large quantities of multivariate data (in the form of n-dimensional images) can be acquired in plasma diagnostics. Visualization techniques have been valuable in understanding such data.

An overview of the data visualization techniques that are available to analytical chemists was presented. The examples included fly-by animations of representations of multivariate data, stereoscopic visualization of three-dimensional data, volume rendered images of tomographic data, and the use of false colours to enhance the informing power of computer images. The use of visualization techniques for the interpretation of plasma diagnostic data were emphasized.

---

### LIMS AND CIM: INTEGRATING THE LAB, THE PLANT, AND THE CORPORATION – CASE STUDIES

*John T. Bianculli, Brian J. Levey and David Wickham, Beckman Instruments, Inc., Laboratory Automation Operations, 90 Boroline Road, Allendale, NJ 07401*

Implementation of computerized automation systems has been growing within production facilities in order to improve departmental efficiency. For example, quality control (QC) laboratories have implemented LIMS (Laboratory Information Management Systems), the production floor has implemented MRP (Manufacturing Resource Planning), administration has implemented order entry, accounting and shipping/receiving systems. In addition, the corporation usually has one or more centralized databases that contain summary data from a number of departments which can be accessed to the appropriate levels by all users.

These individual systems have truly been effective within the departments. Historically, these systems have been individually justified and acquired based on the quality and productivity benefits that the given system could provide. Improvements in software capability, and enhancements in computing and network technology, have made greater interoperability of these individual systems possible.

In order to gain a competitive edge, progressive companies have advanced to another level of automation by joining systems to share the appropriate information between systems and departments. The joining of the various departmental systems into a single enterprise is often referred to as CIM (Computer Integrated Manufacturing).

Case studies were presented to illustrate how LIMS Systems have been incorporated into CIM Systems. The objective is to provide quality and productivity gains through automated interaction between various plant automation software systems. This results in a more unified and cohesive enterprise-wide automation system, rather than discreet departmental islands of automation.

---

### TOTAL INTEGRATED MANUFACTURING (TIM) – THE ANALYSIS AND DESIGN OF FULLY INTEGRATED MANUFACTURING SYSTEMS IN REGULATED ENVIRONMENTS

*S. P. Maj, Institute of Automatic Control Systems, Servolaboratoriet, Technical University of Denmark (DTH), Building 326 DK-2800, Lyngby, Denmark*

All organizations should have an information strategy and hence a long-term view to prevent the fragmented and uncoordinated introduction of computer based systems. With this impetus it is then possible to define a Corporate Data Architecture. The technology employed should then be part of a computer based system that integrates, both vertically and horizontally, the total manufacturing complex. In this context a major problem in the industry today is the design, implementation and maintenance of Laboratory Information Management Systems (LIMS) in regulated environments. The development of computer based systems in these environments must address the problem of quality. Quality must be demonstrably built into the system. Unfortunately it is the common experience to be given responsibility without guidance other than regulations. Resulting inadequate attention to the analysis and design of data structures and the associated data processing found in laboratories prior to the introduction of a Laboratory Information Management Systems (LIMS) can result in a computer system that does not fully satisfy user requirements. The resulting system may be inefficient and inflexible, or even error prone with 'open ended' maintenance. What is needed is a method to address these strategic, tactical and operational issues with clear guidelines, employing proven techniques and intrinsic documentation in order to fully identify, map, validate and optimise the required functions, events and data. In a highly regulated environment this would help ensure verification and validation to the required standard.

---

### ON-LINE PROCESS CONTROL OF INORGANICS WITH NIR

*Karen Oder, Solvay, Deutsche Solvay-Werke, Xantener Strasse 237-57, Postfach 1351 & 1361, D-4134 Rheinberg 1 Germany; and Cynthia Kradjel and Ulrich Meyhack, Bran + Luebbe, Werkstrasse 4, D-2000 Norderstedt, Germany*

The majority of technical literature on Near Infra-red technology discusses analysis of (1) hydrogenic stretches of organic compounds (C–H, O–H and N–H); or (2) moisture.

Although not widely known, this technology can also be applied for process control of inorganics. Near infra-red analysis has been successfully installed at Solvay since 1984 for the determination of inorganics. The technology is used to monitor the ammonium-soda process, known as the Solvay process, that has the following chemical reaction:

$$2\,NaCl + CaCO_3 \rightarrow Na_2CO_3 + CaCl_2$$

Constituents currently analysed are chloride, carbonate, total ammonium total alkalinity and sodium alkalinity. Eight systems are installed in-process and linked by a PC-workstation for the processing of data. Performance of the entire NIR network is verified by a central Near Infrared analyser located in the laboratory.

Since the network is installed, a stabilization of the process is obvious.

---

### ON-LINE GRADIENT CALIBRATION IN INDUCTIVELY-COUPLED PLASMA EMISSION SPECTROMETRY

*Timothy K. Starn, Norman N. Sesi and Gary M. Hieftje, Department of Chemistry, Indiana University, Bloomington, IN 47405*

Sample automation is a central theme in the development of modern analytical instrumentation. Autosamplers have been used for some time, but very few methods exist for automatically and continuously generating and introducing calibration standards. Accurate and reproducible standards are difficult to obtain via mechanical means. Active control of the standard-producing process is required, making a microprocessor the logical choice for overseeing this control.

Modern, high-performance liquid chromatography (HPLC) pumps are already in an advanced stage of microprocessor integration. An HPLC pump was used to produce calibration curves in inductively-coupled plasma-optical emission spectrometry (ICP-OES). Detection limits for Ca, Y and Cu standards made with programmed gradients were found to be comparable to, or better than, calibrations performed with individually prepared standards.

The on-line gradient technique offers programmable ease-of-use and permits the automated selection of analytes and blank solutions. Another advantage the method enjoys is computer-generated spiking for doing standard additions. The advantages and disadvantages of gradient calibration were discussed as was the future of this automating application.

---

### A FULLY AUTOMATED AND VERSATILE FUSION SYSTEM FOR RAPID SAMPLE PREPARATION: SOLUTIONS AND X-RAY BEADS

*Scott W. McGeorge, Leco Instruments Limited, 5151 Everest Drive, Mississauga, Ontario, Canada L4W 2R2*

The principles and analytical merits of automated fusion systems for the rapid digestion of a wide range of sample matrices were presented. The FX-503 Fluxer is capable of high temperature borate-type fusions or low temperature peroxide fusions. A very wide range of agitation modes can be programmed in concert with automated non-wetting agent dispensing and ultimate pouring of the melt into solution beakers or casting dishes. For solution preparations, the beaker stages permit magnetically coupled stirring with heating via a hot plate. Casting dishes receive the melt for glass bead formation after the crucible has been heated on the pouring side, and subsequent cooling of the bead is controlled by an adjustable compressed air flow.

Parameters that can be programmed by the operator for each phase of the method include: burner temperature, oscillation frequency, oscillation amplitude, non-wetting agent addition, heating/cooling of the casting dish, and the preparation time period for each phase. Up to 99 different methods can be stored on the internal computer system.

Several new features enhance the production capacity of the system and simplify operation to provide a cost-effective and safe alternative to the processing of difficult samples.

---

### IMPROVEMENTS IN AUTOMATIC GEOLOGICAL MATERIAL DIGESTION USING A FOCUSED OPEN VESSEL MICROWAVE DIGESTER FOR TRACE METAL ANALYSIS

*A. M. de Kersabiec, F. Vidot, J. Boulegue, Laboratoire de Géochimie et Métallogénie (CNRS URA 196), Université Pierre et Marie Curie, 4 place Jussieu, 75252 Paris, France; and I. Verhaegue, Instruments S.A. division Jobin-Yvon, 16/18 rue du Canal, 91160 Longjumeau, France*

The traditional hot plate digestion techniques to dissolve geological materials for trace metal analysis are often time consuming, tedious and subject '◦ operator errors. These techniques usually take up to 16 hours, require evaporation to near dryness to remove silicates and require re-heating of the residue to effect solution.

The introduction of microwave digestion systems has totally transformed the hot plate digestion technique. The method developed and discussed here for the preparation of geological samples used the Prolabo Microdigest 301, an open vessel focused microwave digestion system This system includes three PTFE pumps for the automatic addition of acids, as well as programming of time and power conditions. It is capable of automatic evaporation to near dryness to complete the digestion process.

The Prolabo Microdigest 301 system has been used in this laboratory for the successful digestion of geological standard reference samples: biotite, basalt, bauxite, granite (CRPG France), soil (USGS), river sediment (BCR). The time required for the digestion of each sample was reduced to approximately 1 h using additions of $HNO_3$/HCl/HF and the completeness of digestion was confirmed by atomic spectroscopy (ICP-AES and AA) techniques. The results agreed well with the certified results for non-volatile elements including As, Cd and Pb.

A special study using the Prolabo Microdigest 301 for the preparation of lead and zinc ores (BCR) demonstrated the efficiency of this system to completely dissolve these materials and to retain traditionally volatile elements such as As, Ge and Sb.

The Prolabo open vessel microwave digestion technique has improved the precision of analysis due to less operator 'ntervention, required less time to complete digestions and demonstrated its usefulness for a wide range of applications.

---

### NEW DEVELOPMENTS IN AUTOMATIC HEADSPACE ANALYSIS

*A. D. Bashall, Fisons Instruments, 32 Commerce Center, Cherry Hill Drive, Danvers, MA 01923; and F. Munari, Carlo Erba Instruments, Strada Rivoltana, 20090 Rodano, Milan, Italy*

Headspace analysis has long been accepted as an excellent method for the analysis of volatile components in complex or dirty matrices. However, problems have existed in method development due to fairly complex investigations into the various factors affecting headspace analysis, in particular the equilibrium time and temperature values as well as the deterimental effects of the application of heat for too long a time.

The system proposed enables the samples to be handled exactly the same way in respect of equilibration time, temperature etc. The sample vials are handled robotically to ensure that each sample is handled exactly the same way, if that is required. The robotic application also allows the method to be developed using an application development program (ADP). The ADP carries out optimization of such conditions as sample volume, incubation time and incubation temperature.

Novel applications of headspace analysis were discussed, in particular the analysis of trace level components by repetitive injections of the same sample. By the use of a highly efficient cold trap multiple injections are concentrated on the head of the column by employing the cold trapping effect. The cold trap then allows extremely rapid heating, typically −150 °C to 250 °C in 17 s, to ensure the concentrated sample leaves the cold trap in one homogeneous plug, thus maintaining chromatographic integrity.

A wide range of applications was discussed.

---

### TOWARDS A FULLY AUTOMATED FOCUSED MICROWAVE DIGESTION SYSTEM

*Max Feinberg, Carolle Suard and Jayne Ireland-Ripert, Institut National de la Recherche Agronomique, Laboratoire de Chimie Analytique, 16, rue Claude Bernard, 75231 Paris 05, France*

Focused microwave digestion is a well established and promising sample preparation technique. However, its diffusion among analysts may be limited because it is necessary to develop totally new operation procedures in order to implement this technique in a laboratory. Thus, full automation cannot consist only in designing automated sampling devices or computer driven instrument control. These facilities are useful, but analysts also require precise guidelines for selecting digestion conditions and designing the heating program according to the matrix type.

The selection of digestion conditions is made possible by using an *expert system approach*, which consists in helping the user determine the major characteristics of the digestion program: reagent type, step number and time-power diagram. The objective of this system is to provide the analyst directions by two means:

(1) A reference protocol defined according to matrix type, analyte and analytical method.
(2) Advice on optimization methods, which permit the analyst to adjust the protocol to his precise needs.

In order to define these precise guidelines, an *empirical modelling approach* was used. This allows a better adaptation of the digestion program to the exact nature of the sample matrix: the optimization step. This step consists in seeking operating conditions that give an optimal value for the analytical response, using mathematical models. A preliminary study was performed on food samples, using Kjeldahl nitrogen determination as a starting point. This approach allows reference protocols to be established for different food matrices; these protocols are stored in a database and can be extracted.

In addition, the system will evaluate the characteristics of a digestion procedure that is not already stored in the database.

Using the experience and the reference protocol acquired in the empirical approach, rules are written to constitute the knowledge base of the expert system. Through these rules, the reference protocol can be extracted from the database. In order to complete the expert knowledge, some thermodynamic characteristics of MICRODIGEST A-301 (Prolabo trade name) were modelled. All knowledge was organized within a frontal expert system interfaced with the digester control program.

### IN-LINE ANALYSIS USING FIBRE OPTIC PROBES FOR MONITORING FAT AND MOISTURE IN CREAMERY BUTTER

*Paul C. Entrop and Frank Dethomas, NIRSystems, Inc., 12101 Tech Road, Silver Spring, MD 20904*

Near infra-red represents one of the leading rapid analytical techniques used for quantitative analysis of basic functional groups found in all food products. in the analysis of Fat and Moisture in creamery butter, a near infrared on-line system equipped with fibre optics transmission paired probes (1-cm pathlength) were used in an open-loop process system and monitored within the spectral regions of 400–1100 nm.

The NIR absorbance spectrum for the butter samples displayed independent absorbance bands for the long chain C–H groups for fat and for the strongly absorbing H–O–H groups for water. Both bands absorbed independently which provided the user with the ability to monitor these two functional groups. Robust calibrations were developed for each component using the linear least squares math treatment contained in the NSAS software.

The objective of this paper was to review the sample presentation method along with the calibration results derived from the on-line monitoring of fat and moisture in creamery butter.

### AUTOMATED SAMPLE FRACTIONATION USING SOLID PHASE EXTRACTION AS PART OF AN INTEGRATED SYSTEM FOR THE IDENTIFICATION OF ANTITUMOR COMPOUNDS IN FERMENTATION BROTHS

*C. Shumate, Hamilton Company, Reno, Nevada; and D. J. Hook, J. J. Yacobucci and J. Guss, Bristol-Myers Squibb Company, Wallingford, CT 06492*

A highly effective automated method for the screening of fermentation broths for biologically active compounds was presented. The broths are separated by polarity into four fractions using solid phase extraction cartridges. The automated system can use up to 48 cartridges which can be positioned over six separate collection racks. A robotic liquid handling system performs the column conditioning, sample and solvent addition, positive pressure elution, and the movement of the cartridges over the collection tubes. The system allows precise control of sample and solvent elution rates and volumes to prevent drying of the sorbent bed. The broth is fractionated on reversed phase columns according to lipophilicity using stepwise elution with successively higher percentages of acetonitrile in water. The resulting fractions are automatically diluted and tested for bioactivity. Those indicating positive results are subjected to HPLC with UV-Vis diode array detection in order to identify the bioactive components. Further physicochemical analysis on the samples is also possible.

### SAMPLE PREPARATION OF GRAIN, NUT AND DAIRY PRODUCTS FOR AFLATOXIN TESTING USING AN AUTOMATED WORKSTATION AND IMMUNOAFFINITY COLUMNS

*Lyn Jordan, Zymark Corporation, Inc., Zymark Center, Hopkinton, MA 01748; and Kevin F. Donahue, Vicam L. P., 29 Mystic Avenue, Somerville, MA 02145*

Aflatoxins are naturally occurring carcinogens produced by moulds found in grains, nuts, and other commodities. They are also found in dairy products as a result of contaminated dairy feeds. Since it is a natural toxin, careful monitoring of these commodities is necessary to protect the food supply from contaminated lots. Contamination can occur in the field, in harvest, and in storage, so there are several points in the process and handling of these foodstuffs where aflatoxin testing is critical.

Laboratories performing aflatoxin analysis must obtain accurate and reproducible data due to the serious health risks and economic losses associated with aflatoxin contamination. Safe handling of potentially carcinogenic samples is also a concern.

The immunoaffinity column used for automation utilizes monoclonal antibodies with a high affinity for Aflatoxins B1, B2, G1, and G2, bound to an agarose gel. This method has received official first action approval by the AOAC.

This paper illustrated the conversion of a manual sample preparation method to an automated sample preparation workstation. One feature of the automated method is the ability of the workstation to make direct HPLC injections, eliminating another sample handling step. Data were presented showing the reproducibility resulting from automating this method. By automating the method, the user reduces the risk of technician-to-technician variations, and reduces the exposure to potentially contaminated samples, minated samples. The consistency of the results achieved can lead to high levels of confidence in producing a quality food product.

### AUTOMATED DISSOLUTION TESTING SYSTEM WITH ABILITY TO RAPIDLY CHANGE FROM UV TO HPLC ANALYSIS

*William A. Hanson and Royal Hanson, Hanson Research Corp., 9810 Variel Ave., Chatsworth, CA 91311; and Jeanne B. Li, Millipore, Waters Chromatography Division, 34 Maple St (TG), Milford, MA 01757*

Development of new oral and transdermal pharmaceutical dosage forms requires multiple dissolution testing. Recent emphasis on more extensive bio-availability data has increased the number and sophistication of the dissolution tests required.

Method development may choose from several analytical procedures, but UV absorbance and HPLC are most generally selected. The number of tests required and time duration, in case of modified release, has made automated systems almost mandatory because of the time and personnel constraints.

Existing automated dissolution systems are widely available for UV absorbance, but separate sampling apparatus is currently needed for HPLC. Investment in two totally separate automated systems is thus currently necessary.

This paper gave details of a simple automated dissolution assembly that can be rapidly converted from UV absorbance to HPLC analytical methods with minimum change-over time. The computer driven system can provide repeatable results with full data reduction of full statistical analysis and printed experimental protocol of either UV or HPLC methods. Examples of dissolution runs using both analytical methods were reported.

---

### A SYSTEM FOR AUTOMATED ANALYSIS OF PHARMACEUTICALS

*Alger D. Salt, Rick T. Davis and David D. Elks, Burroughs Wellcome Co., Analytical Development and Quality Assurance Laboratories, Greenville, NC 27834*

Five Zymark robotic dissolution systems, two Zymark Pysystem sample preparation robots, and six Zymark BenchMate WorkStations are now used for sample preparation of various pharmaceutical formulations in the Quality Assurance and Analytical Development Laboratories at Burroughs Wellcome Co. In 1990 over 22 000 samples were processed on the dissolution system and 3300 samples on the sample preparation robots. Similar sample volumes on the more recently installed BenchMates in the coming year are expected.

The three different types of robotic units operate independently. Each system performs a particular task within a scheme for automated sample analysis as shown below. Solid dosage form samples are dissolved or disintegrated on either a dissolution system or a sample preparation robot. The resulting solutions are placed in test tubes on racks. If the solutions are ready for analysis, the rack is taken to the appropriate analytical instrument. If more sample processing is required, the rack is taken to a Zymark BenchMate Workstation for final liquid handling operations such as dilution, mixing filtration, and introduction to either an ultraviolet (UV) spectrometer or a high performance liquid chromatograph (HPLC). Liquid formulations can be processed directly and entirely by the BenchMate. This sample analysis scheme is modular and flexible, allowing lab managers to make the best use of available instruments and analyst time.

This presentation included a discussion of problems and solutions encountered in implementing the sample analysis scheme. Much of the talk focussed on what is done to ensure that the systems are and remain compliant.

---

### AUTOMATION AND IMPROVEMENT OF SOLID-PHASE EXTRACTION (SPE) IN HPLC USING A NEW ON-LINE SAMPLE PREPARATION INSTRUMENT OSP-2

*Annette Knüppel, Manfred Wotschokowsky and Martina Witzenbacher, E. Merck, Frankfurterstrasse 250, D-6100 Darmstadt, Germany*

The new OSP-2 device integrates solid phase extraction on-line into the HPLC system, thus automating the complete analysis from injection to data processing. Solid phase extraction (SPE) is used successfully as a sample preparation method for removing interfering matrix components and concentrating the analytes under investigation. However, the principle has been optimized and has led to the development of the OSP-2 instrument, which allows full automation and integration of solid-phase extraction into the HPLC process. High quality slurry-packed extraction cartridges guarantee high reproducibility, no sample loss and unattended operation. The work described two applications demonstrating the reproducibility, and efficiency of SPE with OSP-2 and newly designed extraction cartridges. The first example was the determination of the anabolic drug stanozolol from plasma. Stanozolol was used illegally for doping in sport and horse racing. The recovery yields were 99·3%, the reproducibility was excellent—between ±0·7 and ±1·3% using the OSP-2 instrument.

The second application presented was the enrichment and automatic determination of pesticides from drinking water with the OSP-2. Only 50 ml water were necessary to gain high sensitivity. One analysis is finished within 2 h.

---

### AN AUTOMATED SOLID PHASE EXTRACTION, HPLC/MS QUANTITATIVE ASSAY FOR SC-50267 (A RENIN INHIBITOR) IN RAT PLASMA

*Min S. Chang and Grant L. Schoenhard, G. D. Searle & Co., Skokie, IL 60077*

An automated solid phase extraction, LC/MS method for the analysis of SC-50267, a peptide mimetic renin inhibitor, in rat plasma (0·4 ml) was developed. The sensitivity of the assay was 25 ng/ml and the assay was linear to 1625 ng/ml. SC-48473, an analog, was the internal standard. The precision of the assay ($N = 4$) was 37·9%, 12·2%, and 14·9% and the analytical recovery was 85·5%, 89·6%, and 86·3% for 30 ng, 100 ng, and 400 ng SC-50267/ml, respectively.

After the addition of internal standard, SC-50267 and SC-48473 were extracted onto the C-18 100 mg BondElut cartridge. The cartridge was washed with water and acetonitrile and dried by an air purge. The SC-50267 and SC-48473 were eluted with triethylamine:formic acid: methanol (0·1:0·1:100, by volume) and evaporated to dryness. The solid phase extractions were performed unattended on a MilliLab which was adapted to use syringe type extraction cartridges. A VG Trio-2 mass spectrometer equipped with a thermospray interface tuned to detect the molecular ions (MH) was used for the detection. A polymeric column (BioGuard C-4, 4 mm × 2 cm, Interaction) cartridge was used to resolve the coeluting analyte peaks from the injection baseline flacuration at the solvent front. The HPLC mobile phase was water : methanol : triethylamine : formic Acid (80 : 20 : 0·75 : 0·5, by volume).

### NEW AUTOMATED SUPERCRITICAL FLUID ANALYSERS FOR IN-FIELD ENVIRONMENTAL MONITORING AND IDENTIFICATION

*S. A. Liebman, Geo-Centres, Inc.; and S. M. Lurcott, S. Sarner and E. J. Levy, Computer Chemical Systems, Inc., Box 683, Avondale, PA 19311*

Environmental monitoring and trace organic analysis of air, water, and hazardous wastes require a range of analytical tools and methods. Development of highly automated instruments based on supercritical fluid (SF) technology has been focused on providing in-field qualitative/quantitative analytical information in a rapid, reliable manner.

CHAMP, the *Chemical Hazards Automated Multimedia Processor,* is a product line designed with dual SF extractors for processing of air sampling cartridges sorbent beds (charcoal, Tenax, Carbosieve, etc.) and soil/solid samples. Method development involves detection and identification of target organics, such as hydrocarbons, priority pollutants, or simulants (pesticides) for threat agents in military applications. Chromatographic and detector components in the system are appropriate to the analytical needs. Liquid carbon dioxide is the mobile fluid, used with or without modifiers and at varied extractor temperatures for enhanced SFE efficiency.

A specialty CCS system, CHAMP-HC, was designed and prototyped for on-site analysis of hydrocarbon (HC) contaminated soils from leaking underground storage tanks. The unit was designed as a compact (ca. 2′ × 2′ × 2, 50 lb) and suitable for a mobile lab facility. Results from method development were obtained using a prototype CHAMP-HC configuration for rapid (ca. 20 min) automated SFE-capillary GC/FID analyses of gasoline and oils contaminated sand, and soils, and clays. Unique data management with expert system and neural networks was explored. Trial runs with commercial neural network software (NeuroShell) provided identification and classification of test gasoline/diesel datasets. The highly automated systems were designed for use with minimal operator training to provide direct readout from ppb to percent levels.

### INTERFACING LABORATORY ROBOTICS WITH OFF-THE-SHELF SOFTWARE

*Joseph C. Roark and Arthur Schleifer, Hewlett-Packard Co., Scientific Instruments Division, 1601 California Ave., Palo Alto, CA 94304*

Just as the ability to interface with standard laboratory hardware enhances the use of the HP ORCA Laboratory Robotic System, the ability of the robot controlling software to interface with off-the-shelf software enhances the integration of laboratory robotics into other parts of the analytical laboratory. Using this software, the Hewlett-Packard Methods Development Software (MDS), tight integration of laboratory robotics systems with spreadsheet, graphics, word processing, and data base programs to provide total automation is easily accomplished.

Through the use of Microsoft Windows Dynamic Data Exchange (DDE), the MDS software is able to exchange data, advise on data, and control other software applications. In addition, MDS can simultaneously provide server functionality using DDE, which allows other client applications to exchange data and control the execution of the robotic system. For example, expert systems software may be used to control MDS and modify the conditions and steps taken during sample preparation in order to optimize a sample preparation procedure. By using MDS with software products that transparently extend DDE communications over industry standard RS232 and local area networks (LAN), laboratory robotics may be integrated into the network of laboratory information, management, and sample tracking.

An example of a robotic bench management system which uses several readily available software products, was used to illustrate the concept of integrating laboratory robotics with off-the-shelf software. This system provides easy user-interface customization, access to local and remote databases, and integration of analytical instruments with the HP ORCA Laboratory Robotic System.

### ROBOTICS AND METHOD DEVELOPMENT – APPLICATIONS TO SOLID PHASE EXTRACTION

*Nigel Simpson, Varian Sample Preparation Products, 24201 Frampton Ave., Harbor City, CA 90710; and Lynn Jordan, Zymark Corporation, Zymark Center, Hopkinton, MA 01748*

Solid Phase Extraction (SPE) is rapidly replacing many of the traditional liquid/liquid extractions in the analytical laboratory. This technology has the advantages of greater selectivity, lower solvent usage and opportunities for batch processing. It may also be automated, and various robotic systems are now available for direct online elution/injection onto HPLC columns, and for the whole extraction process.

The 'data trail' left by a robotic system such as the Zymark Benchmate provides defensibility for each extraction performed, recording volumes or weights of each step in each extraction. However, its potential for tracking SPE method development has often been overlooked. The Benchmate system is employed with Varian's Bond Elut Certify II extraction cartridge (a sorbent dedicated to extraction of acidic and neutral drugs from biological fluids) to assess the ruggedness of an extraction, and to investigate the effects of changing the extraction variables such as elution solvent volume. The advantages of having precisely reproduced conditions for the other variables such as flow rate and solvent volumes, as confirmed by the data trail left by the robot, were emphasized.

Recovery of the drug metabolite (THC-COOH) was demonstrated for the experiments carried out, and a description of the exact nature of the extraction process were given. The final method was analysed for its reproducibility. This method has been shown to permit quantitation down to 2·5 mg/ml of urine, and over a linearity range of 10 to 30 ng/ml at greater than 85% recovery. Description of how to transfer this to other means of execution for routine analysis concluded the presentation.

---

### ON-LINE MICROWAVE SAMPLE DIGESTION FOR DIRECT ATOMIC SPECTROSCOPIC ANALYSIS

*S. J. Haswell and D. A. Barclay, School of Chemistry, University of Hull, Hull HU6 7RX, UK*

Microwave dissolution techniques are now becoming established methodology for the preparation of a wide range of sample matrices suitable for elemental determination by atomic spectroscopic techniques. The current microwave methodology based on batch or discrete digestion of samples, whilst offering more rapid digestion times and solubilization of difficult matrices over traditional techniques, still require laborious sample handling and volumetric preparations. In addition the so called bomb or vessel digestion techniques are still inherently difficult to control in respect of contamination and real on-line process analysis.

This paper described the development of flow injection analysis technique directly coupled for solid slurry samples which incorporates on-line microwave digestion with element specific detection, so offering a rapid dissolution and detection technique with process control characteristics. The discussion included details of the parameters optimized in developing the technique and consider the mechanisms that may be involved in this rather novel reaction system.

Results obtained using the system developed were evaluated for their precision and accuracy, and included a range of elemental determinations for a selection of solid environmental type samples. A critique of the advantages and disadvantages of the on-line digestion approach was given and compared to dissolution and ablation methodologies currently used or being developed.

---

### TOTAL PHOSPHATE DETERMINATION IN WASTEWATERS BY ON-LINE MICROWAVE DIGESTION INCORPORATING COLORIMETRIC DETECTION

*K. Williams, S. J. Haswell and D. A. Barclay, School of Chemistry, University of Hull, Hull HU6 7RX, UK; and G. Preston, Severn Trent Laboratories, Birmingham, UK*

Wastewater samples, which often contain high levels of suspended organic solids, are routinely analysed for phosphate concentration. This form of routine analysis requires rapid, simple and robust methodology in order to handle the large numbers and varying concentrations of phosphate found in such samples. Sample dissolution has been carried out traditionally by open vessel heating after the addition of $HNO_3$ and more recently by batch microwave digestion. Once dissolution is accomplished various amperometric and colorimetric flow injection systems for subsequent detection of phosphate have been employed.

This paper described a flow injection system for on-line microwave digestion of wastewater samples with total phosphate determination by means of colorimetric detection. Acidified samples are introduced into a water carrier stream and digested under pressure in a microwave cavity under continuous flow conditions in thin bore tubing. Following digestion the samples are cooled on-line and then combined with further reagent streams for subsequent colorimetric detection based on the formation and reduction of heteromolybdophosphoric acid to molybdenum blue as an FIA peak at 660 nm. Signals from the flow through detector are recorded as peak height on a chart recorder. Optimization of parameters such as digestion tube length, diameter and reagent concentrations were discussed. Calibration has been found to be linear to 40 ppm phosphate with a limit of detection of 0·25 ppm. Samples of wastewater can be analysed at a rate of 30–40 seconds per sample with typical sample RSD's of <5% being achieved. Percentage recoveries can be expected to be in the range 95–105%. Results for a range of samples have been found to agree with those obtained by a 'block' digestion autoanalyser method.

---

### AUTOMATED MEASUREMENT OF LOW LEVEL PHENOLS USING FLOW ANALYSIS TECHNIQUES WITH A HIGH PERFORMANCE ABSORBANCE DETECTOR

*Richard J. Berman and Shirley F. Arment, Alpkem Corporation, 9445 S.W. Ridder Road, Suite 310, Wilsonville, OR 97070*

Recent mandates by regulatory agencies have targeted phenol as a priority pollutant. This further emphasizes need for a simple and reliable method for the determination of very low levels of phenols in water and wastewater. Water containing phenols in the low parts per billion range can result in the production of undesirable derivatives upon purification. While much pollution control equipment has been installed to reduce the amount of phenols discharged, the actual amount release is unknown if the analytical method is insensitive or unrealiable at very low levels.

Current standard manual methods are either insensitive when measured directly on a distilled sample, or even more labour intensive when a chloroform extraction is required on the distillate to achieve low ppb detection levels. Current automated methods simplify the analysis but do not reliably measure phenols below a few ppb.

An automated method capable of measuring very low level phenols is presented which uses a high performance absorbance detector. In addition, other flow techniques, such as the use of membranes, preconcentration columns and flow injection analysis have been investigated in order to enhance the sensitivity to phenols.

---

### OVERCOMING DIFFICULTIES OF HIGH BACKGROUND ABSORBANCE IN AUTOMATED FLOW ANALYSIS

*Shirley F. Arment, Curtiss N. Penn, David R. Christmann and Richard Berman, Alpkem Corporation, 9445 S.W. Ridder Road, Suite 310, Wilsonville, OR 97070*

Automated flow analysis is widely used for clinical, environmental, food, agricultural, and industrial product testing for both raw and finished materials. One of the problems associated with flow analysis and varying sample matrices is the presence of unwanted reagent absorbance, sample colour, or sample turbidity, which lead to difficulties for absorbance detection. While automated flow methods have largely replaced labour intensive manual methods to achieve higher sample throughput, the problem of background interference still remains. These difficulties are typically manifested as limited dynamic range, poor analytical precision and accuracy, and inferior detection limits.

A new approach to automated flow analysis can address many of the difficulties associated with reagent and sample absorbance, specifically in wastewater, seawater, and soil samples. The effects of flow dynamics, sample and reagent introduction, wavelength selection, and mathematical treatment of the absorbance data were investigated for overcoming background absorbance and optimized performance of automated flow analysis. Data were presented showing the results of this investigation on low detection limits, accuracy, precision, and sample throughput.

---

### ON-LINE MEASUREMENT OF TOTAL SULPHUR IN REFINERY FUEL GAS

*James S. Wreyford Antek Industrial Instruments Inc., 300 Bammel Westfield Road, Houston, TX 77090*

An on-line analytical method has been developed for the measure of total sulphur in refinery fuel gas. The system can be used to monitor the fuel gas before combustion in refinery applications.

The fuel gas is combusted in a high temperature furnace to convert all sulphur compounds to sulphur dioxide. UV fluorescence detection is utilized to detect and measure the total sulphur content in the sample.

---

### ON-LINE COUPLED HPLC/HRGC: A FULLY AUTOMATIC METHOD FOR THE CHARACTERIZATION OF AROMATIC COMPONENTS IN DIESEL FUELS

*A. D. Bashall, Fisons Instruments, 32 Commerce Center, Cherry Hill Drive, Danvers, MA 01923; F. Munari and F. Andreolini, Carlo Erba Instruments, Strada Rivoltana, 20090 Rodano, Milan, Italy*

Many different analytical techniques have been reported for the determination of polycyclic aromatic components (PAC) in complex matrices such as diesel fuels. Traditional methods of isolating the aromatic fraction of a fuel have included chromatography using silica or alumina adsorbents or solvent partitioning. Further separation and detection/identification has usually been carried out by capillary GC. These multiple stage off-line techniques are often time-consuming and introduce significant errors due to solute loss and contamination during collection and reconcentration steps.

On-line coupling of HPLC/HRGC offers significant improvements over equivalent off-line methods: sensitivity is increased since the whole HPLC fraction is analysed, sample handling is minimized and automated routine analyses are possible. Furthermore automatic online HPLC/HRGC can also be applied to group the PACs by ring size by applying two dimensional LC prior to GC transfer.

A thorough characterization of diesel fuel PAC components are of extreme importance for fuel standardization and environmental impact issues associated with exhaust emissions. PACs are produced during incomplete combustion and since many are known human carcinogens identification and quantitation of these components are of considerable interest.

The chromatographic system described is based on the Dualchrom 3000 HPLC/HRGC instrument employing multidimensional LC coupled to HRGC with MS detection (LC/LC/GC/MS). The addition of the benchtop quadrupole mass spectrometer, Trio-1000, was discussed.

